# Anatomical Variation of the Thoracodorsal Nerve in Extensive Axillary Disease: A Case Report

**DOI:** 10.7759/cureus.85425

**Published:** 2025-06-05

**Authors:** Lily J Owens, Elizabeth Tan, Michael Issac, Peter A Barry

**Affiliations:** 1 General and Breast Surgery, The Northern Hospital, Melbourne, AUS

**Keywords:** axillary lymph node dissection, axillary surgery, rare anatomical variants, thoracodorsal nerve, thoracodorsal pedicle

## Abstract

The thoracodorsal nerve (TDN) is an important anatomical landmark to identify and preserve during axillary surgery. We report a rare case of aberrant TDN anatomy in a 55-year-old female undergoing axillary lymph node dissection (ALND) for locally advanced left breast cancer. Intraoperatively, the TDN was not in its expected position in the axilla and, once identified, was observed passing posterior to the thoracodorsal vascular pedicle. The nerve was separated posteriorly from the vascular pedicle by a large nodal mass and remained posterior until entering latissimus dorsi (LD). This report discusses the current understanding of TDN variants and implications for misidentifying or injuring the TDN during axillary surgery. Understanding these variants is especially important in the climate of de-escalation of surgical management of the axilla in breast cancer.

## Introduction

Surgical clearance of the axilla is indicated in various malignancies, including breast, melanoma, and other rarer cutaneous forms (e.g., cutaneous squamous cell carcinoma, Merkel cell), usually where there is proven axillary metastasis [1-3]. Axillary lymph node dissection (ALND) may pose a significant surgical challenge in patients who have previously undergone axillary surgery such as sentinel lymph node biopsy (SLNB) or who have extensive axillary disease, due to anatomical distortion [4].

Critical motor nerves to identify and preserve during axillary surgery are the thoracodorsal (TDN), long thoracic, and medial pectoral nerves [5,6]. The TDN arises from the posterior cord of the brachial plexus, from cervical spinal roots six to eight and is a pure motor nerve supplying the latissimus dorsi (LD) muscle [7]. During its course in the axilla, the TDN meets and crosses the thoracodorsal vascular pedicle from medial to lateral and travels anteriorly in this bundle to supply LD [6,8]. Variants to this course exist, albeit uncommon, and are important to recognize during axillary surgery to avoid neuropraxia or permanent damage [6]. Awareness of potential anatomical variants may aid in complete surgical clearance by ensuring recognition of key anatomical landmarks in the axilla, as well as preventing injury to axillary structures. We report a rare case of aberrant TDN anatomy during ALND.

## Case presentation

A 55-year-old female presented with a large mass in the left upper outer quadrant of the breast, present for five years, along with clinically extensive left axillary lymphadenopathy. A grade 2, hormone receptor-positive, invasive ductal carcinoma was diagnosed, and a fine needle aspirate of one of the palpable left axillary lymph nodes confirmed metastasis. No distant metastatic disease was evident on the fluorodeoxyglucose positron emission tomography (^18^FDG-PET) staging scan. She was treated with tamoxifen and goserelin preoperatively and underwent a left therapeutic mammoplasty and ALND with a symmetrizing right mammoplasty. Preoperatively she had no lymphoedema but reported intermittent left arm pain. Her past medical history was significant only for type two diabetes mellitus.

**Figure 1:**
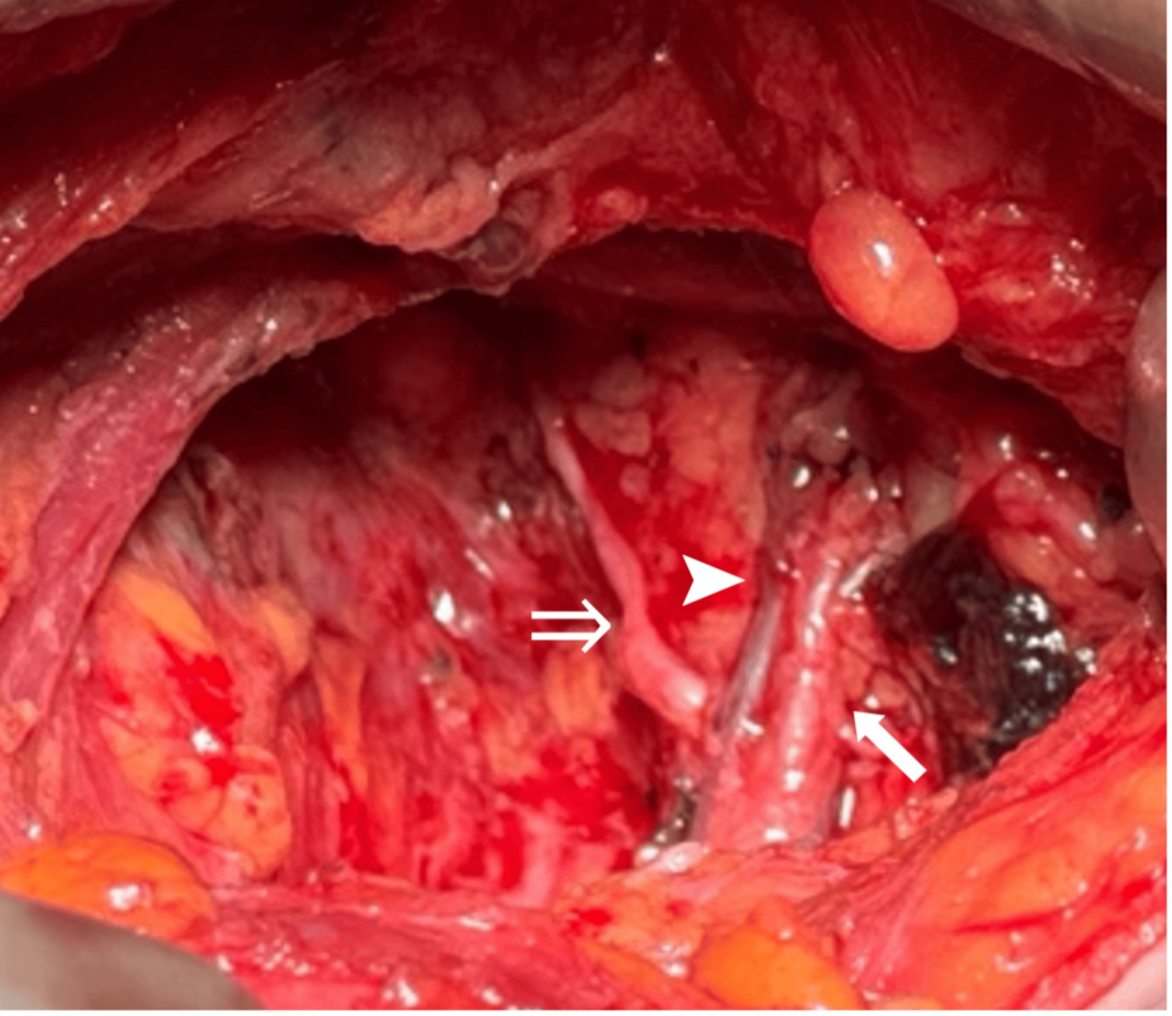
Dissected axilla demonstrating thoracodorsal nerve passing posterior to thoracodorsal artery and vein. Thoracodorsal nerve (⇒); thoracodorsal vein (➤); thoracodorsal artery (⬉)

Intraoperatively, a wide excision of the left breast lesion was followed by ALND. Due to the extent of palpable bulky axillary disease, clearance included level one to three nodes. The axilla was accessed via the mammoplasty incisions. Axillary tissue was carefully dissected to demonstrate the axillary vein superiorly and the long thoracic nerve descending on the lateral chest wall. The TDN was not initially visible in its expected location superiorly, deep to the axillary vein; however, dissection from below (caudad) revealed the thoracodorsal vascular pedicle anterior to lateral nodes with the nerve passing posterior to the mass and to the thoracodorsal artery and vein before entering LD (Figure 1). The nerve remained posterior and medial to the vascular bundle as it entered the muscle. The nerve was confirmed to be the TDN by mechanically eliciting contraction of LD. The nerve was separated posteriorly from the vascular thoracodorsal bundle by the nodal mass, with the latter passing anterior to it. Additionally, the nerve was observed entering the thoracodorsal bundle from the posteromedial aspect, and once joined, continued with the vessels into LD. It was preserved with careful dissection to remove the surrounding axillary tissue.

The patient reported no weakness in the left shoulder girdle or upper limb post-operatively. Recovery was complicated by a left axillary seroma requiring aspiration under ultrasound guidance and a course of oral antibiotics. Chemotherapy, cluster of differentiation (CD)4/6 inhibitor, and endocrine therapy were recommended as adjuvant systemic treatment following surgery.

## Discussion

From its origin in the brachial plexus, the TDN travels down the posterior wall of the axilla, sloping forward to meet the subscapular artery and vein [6-10]. After giving off the circumflex scapular artery, these vessels become the thoracodorsal artery and vein, and this neurovascular pedicle enters and supplies LD [6-10]. Whilst reports of anatomical variants in the axilla exist [11], most mention variations of the brachial plexus and axillary vasculature seen in both donor cadavers and in patients undergoing ALND [12]. Reports of anatomical variation of the TDN focus on its origin rather than its course, and most are cadaveric [13,14]. This is perhaps because the TDN has a consistently described course in the axilla and because variants are relatively infrequent compared to other axillary structures [11].

Standard anatomical texts describe the TDN running anterior and lateral to the vessels in the neurovascular bundle to LD [6-10]. However, rare variants of the thoracodorsal vein, artery, and nerve in the thoracodorsal pedicle have been described. In a study of 63 in vivo ALNDs, Khan et al. identified seven cases in which the TDN arose medial to the vein and artery and eventually passed anterior across the vessels to lie superficial in the pedicle as it entered LD [11]. This group additionally reported three further patients in whom the TDN remained medial and posterior to the vessels until entering LD, comparable to the variant we describe [11]. A similar variant was also described in 1998 by Kutiyanawala et al. in a study of 100 patients undergoing axillary dissection for breast cancer [15].

Injury to the TDN is a serious but rare complication of axillary surgery, resulting in loss of motor supply to LD and subsequent weakening of ipsilateral shoulder adduction and internal rotation [5,6,8]. Correct identification of this nerve during axillary surgery is therefore essential to prevent long-term LD weakness, atrophy, and disability [16].

This case is noteworthy due to the significantly different location of the TDN from its expected position during ALND and the infrequency of such variants in the literature. As the TDN is so consistently taught as passing anterior to the artery and vein prior to entering the thoracodorsal pedicle, the aberrant location in this case posed a challenge in identifying the TDN and distinguishing it from other nerves in the axilla. This demonstrates the importance of awareness of possible anatomical variants when operating, as well as surgeon adaptability in approaching the axilla from different directions (both above and below) when such structures are not seen in the expected location, as in our case. Documentation of these anatomical variants in the literature is therefore necessary to contribute to this understanding and continued safety of surgical practice, especially at a time when de-escalation of more comprehensive axillary surgery means less exposure of trainees and junior consultants to these variations.

## Conclusions

This report details a rare case of TDN course variation in a patient undergoing ALND for locally advanced breast cancer. The TDN was not located in its expected position in the axilla and was challenging to locate intraoperatively. The TDN passed posterior to the thoracodorsal vascular pedicle, contrary to standard anatomical teaching, and was separated posteriorly by a large nodal mass. As a critical motor nerve in the axilla, correctly identifying and preserving the TDN during axillary surgery is essential to protect the normal function of the upper limb and back. Documenting these rare variants in anatomy remains important for the shared knowledge of those operating in the axilla.
